# SARS‐CoV‐2 infection in pregnancy during the first wave of COVID‐19 in the Netherlands: a prospective nationwide population‐based cohort study (NethOSS)

**DOI:** 10.1111/1471-0528.16903

**Published:** 2021-09-26

**Authors:** EM Overtoom, AN Rosman, JJ Zwart, TE Vogelvang, TP Schaap, T van den Akker, KWM Bloemenkamp

**Affiliations:** ^1^ Department of Obstetrics Women and Baby Division Birth Centre Wilhelmina Children Hospital University Medical Centre Utrecht Utrecht The Netherlands; ^2^ Department of Obstetrics and Gynaecology Diakonessenhuis Utrecht The Netherlands; ^3^ Perined Utrecht The Netherlands; ^4^ Department of Obstetrics and Gynaecology Deventer Hospital Deventer The Netherlands; ^5^ Department of Obstetrics and Gynaecology Leiden University Medical Centre Leiden The Netherlands; ^6^ Athena Institute VU University Amsterdam The Netherlands

**Keywords:** coronavirus disease 2019, obstetric surveillance system, pregnancy, pregnancy complications, severe acute respiratory syndrome coronavirus 2

## Abstract

**Objective:**

To describe characteristics, risk factors and maternal, obstetric and neonatal outcomes of pregnant women infected with severe acute respiratory syndrome coronavirus 2 (SARS‐CoV‐2).

**Design:**

Multi‐centre prospective population‐based cohort study.

**Setting:**

Nationwide study in the Netherlands.

**Population:**

Pregnant women with confirmed SARS‐CoV‐2 infection admitted to hospital or in home‐isolation: 1 March 2020 to 31 August 2020.

**Methods:**

Pregnant women with positive polymerase chain reaction or antibody tests were registered using the Netherlands Obstetrics Surveillance System (NethOSS). (Selective) testing occurred according to national guidelines. Data from the national birth registry (pregnant pre‐coronavirus disease 2019 [COVID‐19] cohort) and an age‐matched cohort of COVID‐19‐positive women (National Institute for Public Health and the Environment; fertile age COVID‐19 cohort) were used as reference.

**Main outcome measures:**

Incidence of SARS‐CoV‐2 infection in pregnant women. Maternal, obstetric and neonatal outcomes including hospital and intensive care admission.

**Results:**

Of 376 registered pregnant women with confirmed SARS‐CoV‐2 infection, 20% (74/376) were admitted to hospital, of whom 84% (62/74) were due to SARS‐CoV‐2; 10% (6/62) were admitted to intensive care and 15% (9/62) to obstetric high‐care units. Risk factors for admission were non‐European country of origin (odds ratio [OR] 1.73, 95% CI 1.01–2.96) and being overweight/obese (OR 1.86, 95% CI 1.51–3.20). No maternal or perinatal deaths occurred. Caesarean section after labour‐onset was increased (OR 1.58, 95% CI 1.09–2.28). Hospital and intensive care admission were higher compared with the fertile age COVID‐19 cohort (OR 6.75, 95% CI 5.18–8.81 and OR 2.52, 95% CI 1.11–5.77, respectively).

**Conclusions:**

Non‐European country of origin and being overweight/obese are risk factors for severe course of SARS‐CoV‐2 infection in pregnancy, risk of caesarean section and hospital and intensive care unit admission are increased.

**Tweetable abstract:**

Pregnant women with SARS‐CoV‐2 in the Netherlands show increased hospital/ICU admission and caesarean section.

## Tweetable abstract

Pregnant women with SARS‐CoV‐2 in the Netherlands show increased hospital/ICU admission and caesarean section.

This article includes Author Insights, a video abstract available at: https://vimeo.com/manage/videos/583325455/8fa530cd6e

## Graphical abstract

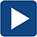

## Introduction

The coronavirus disease 2019 (COVID‐19) pandemic, caused by severe acute respiratory syndrome coronavirus 2 (SARS‐CoV‐2), has had a major impact worldwide.^1^ High‐risk populations have been identified, including the elderly, obese and ethnic minority groups. Evidence is increasingly showing that pregnant women and their unborn children may also comprise a vulnerable group, with higher rates of intensive care (ICU) admission and mechanical ventilation.^2^, ^3^

It is known that pregnant women are not only more frequently affected by pneumonia, but their outcomes are often worse compared with non‐pregnant women.^4^, ^5^ Maternal physiological adaptations in pregnancy, and the physiological state of relative immune suppression, place pregnant women at increased risk of poor outcomes. It is likely that these mechanisms will also play a role in COVID‐19. During previous coronavirus epidemics with SARS‐CoV and Middle East respiratory syndrome coronavirus, risk of maternal mortality and morbidity, as well as miscarriages and preterm labour, were considerable.^6^, ^7^

A meta‐analysis indicated that pregnant women may be at increased risk of ICU admission compared with age‐matched non‐pregnant women and that rates of vertical transmission appear to be very low.^8^ A population‐based cohort of pregnant women with SARS‐CoV‐2 admitted to UK hospitals showed an over‐representation of women from Black, Asian or other minority ethnic backgrounds and those with obesity or pre‐existing medical conditions.^9^ Most studies have only reported on women admitted to hospital, so it is unclear whether the risk of admission itself is increased among pregnant women, and whether findings in terms of risk groups can be generalised to all pregnant women with SARS‐CoV‐2.^8^, ^10^

In the present study, we have collected information on pregnant women with confirmed SARS‐CoV‐2 infection in the Netherlands, both in home‐isolation and admitted to hospital. To provide health professionals with information on SARS‐CoV‐2 and pregnancy, crude numbers of collected cases were previously published on the website of the Dutch Society of Obstetrics and Gynaecology.^11^ In‐depth analysis or comparisons with reference groups are presented here.

The primary aim of this study was to investigate the incidence and maternal, obstetric and neonatal outcomes including hospital and ICU admission and medication use in pregnant women with SARS‐CoV‐2 infection. Secondary aims were (1) to assess factors associated with a more severe course of disease and (2) to explore the effect of pregnancy itself in women of fertile age with SARS‐CoV‐2 infection.

## Method

This is a multi‐centre prospective nationwide population‐based cohort study conducted between 1 March 2020 and 31 August 2020. Cases were ascertained using the Netherlands Obstetric Surveillance System (NethOSS), a nationwide registration system functioning under the umbrella of the Dutch Birth Registry (Perined), in which maternal mortality, severe maternal morbidity and rare diseases in pregnancy are registered.^12^, ^13^ All hospitals in the Netherlands with an obstetrician‐led maternity unit (*n* = 74) were asked to report pregnant or postpartum women up to 42 days with a confirmed SARS‐CoV‐2 infection to NethOSS. All midwifery practices (*n* = 577) were approached through the Society of Midwifery (KNOV), maternity care (*BO geboortezorg*) and Perined and were also asked to report. From March 2020, the joint national guideline on SARS‐CoV‐2 infection in pregnancy of the Dutch Society of Obstetrics and Gynaecology (NVOG), the Royal Society of Midwifery (KNOV) and maternity care (*BO geboortezorg*), the Dutch Society of Paediatricians (NVK) and the Dutch National Institute for Public Health and the Environment (RIVM) indicated that all confirmed cases had to be reported to NethOSS.

In each of the 74 hospitals with an obstetrician‐led maternity unit, a NethOSS reporting physician or midwife was nominated to report cases on behalf of the perinatal cooperation group, based on the organisation of Dutch birth care. Weekly requests were sent by email to reporting professionals. This email contained a reporting link specific to each reporter. Clinicians were asked to report any case meeting the inclusion criteria or reply with '0' if they had no cases to report. For every reported case, information with regard to the woman’s birth year, parity, estimated due date, date of positive SARS‐CoV‐2 test and information on hospital admission, management and birth was provided. Subsequently, a data collection form with additional questions was sent to each reporting physician or midwife. This form was designed by the International Network of Obstetric Survey Systems, based on the UK Obstetric Survey System form with input from the World Health Organization and slightly adapted to the Dutch healthcare system.^14^

For nationwide comparison, two reference groups were established. One reference group consisted of pregnant women pre‐COVID‐19 (pregnant pre‐COVID‐19 cohort) using information from Perined. This registry contains population‐based information pertaining to 99% of pregnancies in the Netherlands.^15^ Specific information of all women in the registry who had given birth between 1 March 2017 and 1 March 2018, the most recent year with complete data, was used. A second reference group consisted of all women of fertile age (20–50 years) with SARS‐CoV‐2 (fertile age COVID‐19 cohort), regardless of pregnancy status, obtained through RIVM. These women were confirmed SARS‐CoV‐2‐positive by polymerase chain reaction or imaging and registered until 31 August 2020. It was unknown whether women were pregnant or not, so this group might also have included pregnant women. The number of ICU admissions was obtained from the National Intensive Care Evaluation, and the number of hospital admissions from the RIVM.

To study the main objective, that is to study characteristics of pregnant women with SARS‐CoV‐2 infection, we described the characteristics of the women enrolled in the NethOSS cohort and compared them with the pregnant pre‐COVID‐19 cohort. To study the secondary aims, which are to assess factors associated with a more severe course of disease, we performed a nested case–control within the NethOSS cohort (in hospital versus in home‐isolation group) and comparison of the NethOSS cohort with the pregnant pre‐COVID‐19 cohort. To explore the effect of pregnancy itself in women of fertile age with SARS‐CoV‐2 infection, we compared the NethOSS cohort with the fertile age COVID‐19 cohort.

As a result of limited testing capacity, the testing policy from 12 March 2020 focused on severely ill people with a suspected SARS‐CoV‐2 infection, high‐risk groups and healthcare staff working during the COVID‐19 pandemic. Pregnant women were at that time not considered a high‐risk group.^16^ Between 12 March 2020 and 30 April 2020, the policy of the RIVM stated that pregnant women required testing only in case of significant symptoms or if hospital admission for SARS‐CoV‐2‐related symptoms was required. Testing capacity was slowly increased and from 30 April 2020 all people were asked to test if they had symptoms related to SARS‐CoV‐2 infection for longer than 24 hours. Because of this change in testing capacity, a sensitivity analysis was performed on the results before and after 30 April. The two available tests in the Netherlands were a polymerase chain reaction using samples taken from the nose and throat, or a serological test based on the presence of SARS‐CoV‐2‐specific antibodies. Neonates born of SARS‐CoV‐2‐positive mothers were tested for SARS‐CoV‐2 if signs or symptoms such as fever or increased infectious parameters were found.

Outcomes collected were signs of pneumonia on imaging, hospital admission, ICU, neonatal ICU or obstetric high‐care admission and administration of pharmacological therapy. We recorded the characteristics of women including body mass index (BMI), age, country of origin, co‐morbidities and gestational age at onset of symptoms. For women who had given birth, mode of delivery, labour induction, analgesia, intrauterine or peripartum transmission and breastfeeding were assessed. Admission to hospital was defined by hospital stay for longer than 24 hours, but women admitted for birth only were not included. Women at birth were divided into a symptomatic and an asymptomatic group. Women were considered symptomatic if complaints related to SARS‐CoV‐2 infection were reported at onset of labour. The incidence of SARS‐CoV‐2 infection was estimated using the most recently available data from Perined in 2018, which included 79 962 pregnancies reported over a period of 6 months.

Country of origin was based on the definition of *Statistics Netherlands*. If the woman was born in the Netherlands with at least one of her parents born abroad, she was considered to be from the same origin as her parent(s) from outside the country. Body mass index was defined according to the first recorded weight in pregnancy up to 12 weeks. Overweight pertained to BMI above 25 kg/m^2^ and obesity to a BMI above 30 kg/m^2^. Gestational age was based on the first‐trimester dating ultrasound.

No core outcome sets were used in this study and the study did not have active patient involvement. No funding was received.

Statistical analyses were carried out using IBM SPSS 25 (IBM, Armonk, NY, USA). Descriptive analyses were performed. Proportions are presented as percentages, and skewed distributions as medians with ranges. For categorical data, differences are presented as odds ratios (OR) with 95% CI.

## Results

Between 1 March 2020 and 31 August 2020, 394 SARS‐CoV‐2‐positive pregnant women were registered. In 18 pregnant women, a positive test result was reported, but additional information could not be retrieved. Additional data were returned for the remaining 376 women (95%). The estimated incidence of SARS‐CoV‐2 among pregnant women in the Netherlands over these 6 months was 4.70 per 1000 maternities. The number of collected cases was highest during the first 2 months of registration (March and April, *n* = 216) with an estimated incidence of 8.10 per 1000 maternities. The number of positive cases per week can be seen in Figure S1. Testing capacity increased after 30 April. In March and April 57/216 (26%) women were admitted, May to September 17/160 (11%) women were admitted. Sensitivity analysis between cases before and after 30 April did not affect our general conclusions.

The majority of pregnant women with SARS‐CoV‐2 were not admitted to hospital (302/376, 80%). Instead, they stayed, as advised by the government, in home‐isolation until symptoms had subsided and 2 weeks after disease‐onset. Of pregnant women with SARS‐CoV‐2 admitted to hospital (74/376, 20%), admission was COVID‐related in 62/74 (85%). Other reasons for admission were signs of imminent premature birth and hypertensive disorders. Six women required ICU treatment, which represents 10% (6/62) of all pregnant women with SARS‐CoV‐2 who were admitted to hospital and 2% (6/376) of all registered pregnant women with SARS‐CoV‐2. Another nine (9/62, 15%) were admitted to obstetric high‐care units with additional monitoring facilities but did not require mechanical ventilation. No maternal death was reported.

Signs of pneumonia on imaging were found in 35/376 (9%) registered pregnant women. They most frequently complained of cough (180/376, 47%), breathlessness (91/376, 24%), flu‐like symptoms (95/376, 25%) and fever (149/376, 39%). Antibiotics were administered in 41/376 (11%) women and antiviral drugs in 5/376 (1%) (oseltamivir, *n* = 2; remdesivir, *n* = 3). In 14/376 (4%), corticosteroids were administered to stimulate fetal lung maturation. This was due to signs of threatened preterm labour (7/14, 50%) or high risk of iatrogenic preterm labour because of the severity of the SARS‐CoV‐2 infection (7/14, 50%). Oxygen supplementation was used in 30/376 women (8%) with signs of breathlessness and low oxygen levels. Four pregnant women (4/376, 1%) needed mechanical ventilation of whom three were ventilated in prone position.

An overview of background characteristics of pregnant women with SARS‐CoV‐2 and comparison with the pregnant pre‐COVID‐19 cohort is shown in Table 1. Table 2 compares pregnant women with SARS‐CoV‐2 requiring hospital admission with pregnant women with SARS‐CoV‐2 in home‐isolation. Among pregnant women testing positive for SARS‐CoV‐2, non‐European women were disproportionately represented in comparison with the pregnant pre‐COVID‐19 cohort (OR 8.96, 95% CI 6.71–10.42) and among pregnant women with SARS‐CoV‐2 admitted to hospital compared with those in home‐isolation (OR 1.73, 95% CI 1.01–2.96). Overweight or obese pregnant women with SARS‐CoV‐2 were also more often admitted to hospital compared with women in home‐isolation (OR 1.86, 95% CI 1.08–3.20). Pregnant women with SARS‐CoV‐2 more often experienced signs of imminent premature labour compared with the pregnant pre‐COVID‐19 cohort (OR 2.37, 95% CI 1.47–3.82). Risk of hospital admission was decreased in the first trimester (OR 0.01, 95% CI 0.00–0.03).

**Table 1:** Background characteristics: pregnant women with SARS‐CoV‐2 versus reference group

Characteristics	Pregnant women with SARS‐CoV‐2 (*N* = 376) *n* (%)	Reference group of pregnant women (pregnant pre‐COVID‐19 cohort)* (*N* = 183 413) *n* (%)	OR (95% CI)** Pregnant women with SARS‐CoV‐2 versus reference group
General			
Age (years)			
<25	26 (7)	16 662 (9)	0.75 (0.50–1.11)
25–30	97 (26)	54 837 (30)	0.82 (0.65–1.03)
30–35	153 (41)	70 615 (39)	1.10 (0.90–1.35)
35–40	71 (19)	34 290 (19)	1.01 (0.78–1.32)
>40	28 (7)	6913 (4)	2.06 (1.40–3.03)
Missing	1	96	
Country of origin			
European	189 (58)	161 464 (90)	0.16 (0.13–0.20)
African	69 (21)	5 (0)	
Asian	19 (6)	7401 (4)	1.44 (0.91–2.29)
South American	5 (2)	4681 (3)	0.58 (0.24–1.41)
Other	45 (14)	6599 (4)	4.17 (3.05–5.72)
Missing	49	3263	
BMI (kg/m^2^)			
Normal (<25)	161 (49)	na	
Overweight (25–30)	100 (30)	na	
Obese (>30)	67 (20)	na	
Missing	48		
Smoking			
Current	16 (5)	na	
Missing	43	na	
Pre‐existing medical problems			
Pulmonary disease	23 (7)	na	
Cardiac disease	6 (2)	na	
Diabetes	6 (2)	na	
Missing	34		
Pregnancy			
Parity			
Nulliparous	159 (42)	79 518 (43)	0.95 (0.78–1.17)
Multiparous	217 (58)	103 549 (56)	1.05 (0.85–1.29)
Missing	0	346	
Trimester at positive test			
First trimester	49 (13)	N/A	
Second trimester	101 (27)	N/A	
Third trimester	200 (54)	N/A	
Postpartum	19 (5)	N/A	
Missing	7		
Multiple pregnancy	9 (2)	5.270 (3)	0.91 (0.47–1.77)
Missing	33	41	
Signs of premature labour	18 (5)	4226 (2)	2.37 (1.47–3.82)
Missing	40	2197	

na, not available.

* Reference group from pregnant women in the Dutch Perinatal Registry (Perined) between 1 March 2017 and 1 March 2018.

** Odds ratio between pregnant women with SARS‐CoV‐2 who have given birth and reference group from Dutch Perinatal Registry.

**Table 2:** Background characteristics: pregnant women admitted to hospital compared with pregnant women in home‐isolation

Characteristics	Pregnant women admitted to hospital (*N *= 74) *n* (%)	Pregnant women in home‐isolation (*N* = 302) *n* (%)	OR (95% CI)* Hospital admission versus home‐isolation
General			
Age (years)			
<25	4 (5)	22 (7)	0.73 (0.24–2.17)
25–30	15 (20)	82 (27)	0.68 (0.37–1.26)
30–35	36 (49)	117 (39)	1.49 (0.89–2.49)
35–40	13 (18)	58 (19)	0.89 (0.46–1.73)
>40	6 (8)	22 (7)	1.12 (0.44–2.87)
Missing	0	1	
Country of origin			
European	32 (47)	157 (61)	0.58 (0.34–0.99)
African	17 (25)	52 (20)	1.33 (0.71–2.49)
Asian	5 (7)	14 (3)	1.39 (0.48 –4.00)
South American	2 (3)	3 (1)	2.59 (0.42–15.79)
Other	12 (18)	33 (13)	1.47 (0.71–3.02)
Missing	6	43	
BMI (kg/m^2^)			
Normal (<25)	26 (37)	135 (52)	0.54 (0.31–0.93)
Overweight (25–30)	25 (36)	75 (29)	1.36 (0.78–2.37)
Obese (>30)	19 (27)	48 (19)	1.63 (0.88–3.01)
Missing	4	44	
Smoking			
Current	8 (11)	8 (3)	4.03 (1.46–11.16)
Missing	3	40	
Pre‐existing medical problems			
Pulmonary disease	6 (8)	17 (6)	1.35 (0.51–3.57)
Cardiac disease	2 (3)	4 (1)	1.90 (0.34–10.59)
Diabetes	1 (1)	5 (2)	0.75 (0.09–6.49)
Missing	2	32	
Pregnancy			
Parity			
Nulliparous	32 (43)	127 (42)	1.05 (0.63–1.76)
Multiparous	42 (57)	175 (58)	0.95 (0.57–1.59)
Missing	0	0	
Trimester at positive test			
First trimester	4 (5)	156 (53)	0.01 (0.00–0.03)
Second trimester	14 (19)	7 (2)	0.56 (0.30–1.05)
Third trimester	44 (60)	*7*	1.31 (0.78–2.19)
Postpartum	12 (16)	7 (3)	7.96 (3.01–21.04)
Missing	0	32	
Multiple pregnancy	2 (3)	6 (2)	1.06 (0.22–5.21)
Missing	1	39	
Signs of premature labour	12 (16)		8.43 (3.04–23.34)
Missing	1		

* Odds ratio between pregnant women with SARS‐CoV‐2 admitted to hospital and pregnant women with SARS‐CoV‐2 in home‐isolation.

Six women had a miscarriage and intrauterine fetal death occurred in one pregnant woman with SARS‐CoV‐2. This was a term pregnancy, and the cause of intrauterine death was unknown. Swabs of amniotic fluid, fetus and placenta were negative for SARS‐CoV‐2.

Information concerning birth was retrieved for 289 pregnant women with SARS‐CoV‐2 (289/376, 77%) and is summarised in Table 3. Results of pregnant women with SARS‐CoV‐2 and a subgroup of those symptomatic at birth (*n* = 70) were compared with the pregnant pre‐COVID‐19 cohort. The risk of caesarean section after onset of labour was increased (OR 1.58, 95% CI 1.09–2.28), especially for women who were symptomatic at birth (OR 2.29, 95% CI 1.20–4.36). Pre‐labour caesarean section was performed in 20 women (20/289, 7%). In only one woman was the indication COVID‐related. Reasons for caesarean section after labour‐onset (32/289, 11%) of pregnant women with SARS‐CoV‐2 were obstructed labour (13/32, 41%), suspected fetal distress (13/32, 41%), both obstructed labour and suspected fetal distress (2/32, 6%) or other (4/32, 13%). Compared with the pregnant pre‐COVID‐19 cohort, labour was more often induced in pregnant women with SARS‐CoV‐2 (OR 4.05, 95% CI 3.18–5.17). The risk of preterm birth (OR 1.01, 95% CI 0.68–1.49) was not increased for all pregnant women with SARS‐CoV‐2 infection; however, it was increased for women who were symptomatic at birth (OR 2.02, CI95% 1.11–3.69).

**Table 3:** Birth characteristics

	Pregnant women with SARS‐CoV‐2 who have given birth (*N* = 289) *n* (%)	Pregnant pre‐COVID‐19 cohort, pregnant women who have given birth* (*N* = 183 413) *n* (%)	OR (95% CI)** Pregnant women with SARS‐CoV‐2 compared with pregnant pre‐COVID‐19 cohort	Pregnant women with SARS‐CoV‐2 who were symptomatic at birth (*N* = 70) *n* (%)	OR (95% CI)*** Pregnant women with SARS‐CoV‐2 who were symptomatic compared with pregnant pre‐COVID‐19 cohort
Mode of birth					
Vaginal birth	227 (79)	123 709 (76)	1.31 (0.92–1.76)	43 (61)	0.50 (0.31–0.80)
Instrumental vaginal birth	17 (6)	12 802 (8)	0.75 (0.46–1.22)	6 (9)	1.09 (0.47–2.53)
Pre‐labour caesarean section	20 (7)	13 477 (8)	0.85 (0.54–1.33)	10 (14)	1.84 (0.94–3.59)
Caesarean section after onset of labour	32 (11)	12 203 (8)	1.58 (1.09–2.28)	11 (16)	2.29 (1.20–4.36)
Missing	8	21 222		0	
Gestational age at birth (weeks)					
16^+0^–<36^+6^	28 (10)	12 352 (10)	1.01 (0.68–1.49)	13 (19)	2.02 (1.11–3.69)
37–40^+6^	196 (72)	80 431 (66)	1.29 (0.99–1.68)	47 (67)	1.05 (0.64–1.73)
≥41	50 (18)	29 009 (24)	0.71 (0.53–0.97)	10 (14)	0.53 (0.27–1.04)
Missing	15	61 639		0	
Induction					
Total	107 (39)	36 885 (22)	4.05 (3.18–5.17)	32 (46)	5.47 (3.41–8.78)
Foley catheter	65 (61)	14 453 (8)	3.16 (2.39–4.18)	18 (58)	3.59 (2.10–6.14)
Prostaglandin	11 (10)	5036 (3)	1.30 (0.71–2.38)	6 (19)	2.96 (1.28–6.84)
Oxytocin/amniotomy	31 (29)	17 396 (11)	1.07 (0.74–1.56)	4 (13)	0.52 (0.19–1.42)
Missing	15	22 024		1	
Analgesia					
Analgesic – opiates	36 (13)	17 314 (9)	0.82 (0.58–1.17)	15 (22)	1.65 (0.93–2.93)
Epidural during labour	72 (25)	32 227 (18)	0.88 (0.67–1.15)	17 (25)	0.89 (0.51–1.54)
Epidural and analgesic – opiates	11 (4)	na		2 (3)	
Missing	3	67 115		3	

na, not available.

* Reference group from pregnant women in the Dutch Perinatal Registry (Perined) between 1 March 2017 and 1 March 2018.

** Odds ratio between pregnant women with SARS‐CoV‐2 who have given birth and reference group from Dutch Perinatal Registry.

*** Odds Ratio between pregnant women with SARS‐CoV‐2 who were symptomatic at birth and reference group from Dutch Perinatal Registry.

An overview of neonatal results can be found in Table 4. No cases of vertical transmission or neonatal death were reported. There were six multiple pregnancies and one intrauterine fetal death, resulting in 295 live births. Of these, 47/295 (17%) neonates were admitted to a neonatal unit. Three neonates were admitted with suspicion of infection. Out of 24 neonates tested; no neonate tested positive for SARS‐CoV‐2.

**Table 4:** Neonatal characteristics

	Neonates of women with SARS‐CoV‐2 (*N* = 295) *n* (%)	Pregnant pre‐COVID‐19 cohort neonates* (*N *= 201 000) *n* (%)	OR (95% CI)** Pregnant women with SARS‐CoV‐2 compared with pregnant pre‐COVID‐19 cohort neonates
Level of care			
No hospital admission	231 (83)	108 106 (70)	2.12 (1.55–2.91)
Neonatal ward	47 (17)	40 675 (26)	0.57 (0.42–0.78)
NICU (total)	7 (3)	6030 (4)	0.64 (0.30–1.35)
Missing	17	46 189	
5‐minute Apgar score			
≤4	2 (1)	2944 (2)	0.55 (0.14–2.21)
5–7	8 (3)	4739 (3)	1.04 (0.51–2.10)
≥8	262 (96)	159 314 (95)	1.26 (0.67–2.38)
Missing	23	34 003	
Perinatal deaths (during labour or postpartum <28 days)	0	121 (0.06)	
Birthweight (median, IQR)***			
Median	3519	3440	
IQR 25	3008	3080	
IQR 75	3762	3775	
Missing	17	16 521	
Culture			
High vaginal tested Positive	21 (9) 1 (5)	N/A	
Amniotic fluid tested Positive	7 (3) 1 (14)	N/A	
Neonate tested Positive	24 (10) 0	N/A	
Missing	50		

IQR, interquartile range; N/A, not applicable.

* Reference group of neonates from pregnant women in the Dutch Perinatal Registry (Perined) between 1 March 2017 and 1 March 2018.

** Odds ratio between neonates of women with SARS‐CoV‐2 and reference group from the Dutch Perinatal Registry.

*** Birthweight in grams.

Hospital and ICU admissions of pregnant women with SARS‐CoV‐2 were also compared with the fertile age COVID‐19 cohort (of whom some were pregnant). Of 19 110 women testing positive for SARS‐CoV‐2 669/19 110 (3.5%) were admitted to hospital and 122/19 110 (6%) to ICU. ORs for hospital and ICU admission were considerably increased: OR 6.75, 95% CI 5.18–8.81 and OR 2.52, 95% CI 1.11–5.77, respectively.

## Discussion

### Main findings

This large nationwide population‐based registration study (NethOSS) provides outcomes of pregnant and postpartum women in the Netherlands who had been infected with SARS‐CoV‐2, during the first wave up to 31 August 2020. No maternal mortality was reported. Among pregnant women with SARS‐CoV‐2 admitted to hospital compared with home‐isolation, those who were overweight and from non‐European countries of origin were over‐represented. Labour induction was more common among pregnant women with SARS‐CoV‐2 and pregnant women with SARS‐CoV‐2 had a higher risk of caesarean section after labour‐onset, especially when symptomatic at birth compared with a pregnant pre‐COVID‐19 cohort. Risk of preterm birth was only elevated for pregnant women who were symptomatic at birth. No vertical transmission was reported. Pregnant women with SARS‐CoV‐2 were at higher risk of hospital admission, especially obstetric high‐care units and ICU compared with a cohort of women with SARS‐CoV‐2 of the same age group (fertile COVID‐19 cohort).

### Strengths and limitations

The prospective population‐based study design with the participation of all Dutch hospitals with an obstetrician‐led maternity unit as well as all midwifery practices and the comparison with data from the national perinatal registry are strengths. We applied the NethOSS registration system that has been in use for nationwide registration of maternal mortality and severe maternal morbidity since 2013. This has resulted in high case ascertainment. RIVM stopped reporting the number of pregnant women positive for SARS‐CoV‐2 after 2 April 2020. They had reported 78 cases by that date, compared with 98 cases reported to NethOSS.^17^ Our results included women with pregnancies of all gestational ages and women admitted to hospital and in home‐isolation with no or mild complaints.

Our study has several limitations. As a result of testing policies, tests were initially limited to people with significant symptoms requiring hospital admission (from 12 March to 30 April 2020). Therefore, some under‐reporting of SARS‐CoV‐2 infections is possible. Moreover, because women with mild symptoms were not always tested and consequently were not included in this study, associations with infection may appear worse than they are. Testing was expanded from 1 May, which is likely to have resulted in more women with mild or no symptoms being included in our study. However, since the number of SARS‐CoV‐2 infections in the Netherlands was generally much lower between 1 May and 31 August 2020, women with mild or no symptoms may still be under‐represented.

We compared the outcomes of pregnant women with SARS‐CoV‐2 with those of a reference group of pregnant women without SARS‐CoV‐2 infection between 2017 and 2018 (pregnant pre‐COVID‐19 cohort), which was the most recent available year with complete data. Recent studies, however, also imply a general effect of the lockdown, for example on premature birth rates and birthweight.^18^, ^19^ We have not been able to assess these general effects in our study.

Registration is still ongoing, and some women analysed in this report were still pregnant at the time of writing (*n* = 81). The effect of SARS‐CoV‐2 on pregnancy, birth and newborns could therefore not be assessed for this group. With a second wave of SARS‐CoV‐2 infection ongoing, it is crucial to analyse perinatal outcomes, including those of women infected in the first and second trimester, because these data have only rarely been reported.^8^

### Interpretation

Even though the majority of pregnant women with SARS‐CoV‐2 infection experienced mild symptoms, a small but significant group developed severe morbidity. This study recognises several risk factors for hospital admission of pregnant women such as increased BMI and non‐European background. When comparing the results of pregnant women to those of all fertile women in the same age group, the pregnant women in our study were more often admitted to hospital and ICU. The reference group contained women up to 50 years of age. As risk of hospital admission is reported to increase with age, and pregnant women in our study population above 40 years of age were scarce (8%) and above 45 years were absent, we assume that the risk could be even higher when compared with women up to 40 years of age.^20^ This reference group will also have included some pregnant women, because pregnancy was not registered by RIVM or National Intensive Care Evaluation. This may have reduced the OR, as pregnant women with higher risk of admission were also included in the reference group. This is the first study to show increased risk of hospital admission in SARS‐CoV‐2‐infected pregnant women in comparison with age‐matched infected non‐pregnant women. Increased ICU admission is supported by recent evidence.^8^, ^21^

The incidence of SARS‐CoV‐2 infection among pregnant women in the Netherlands was higher than reported for the UK, but the UK sample was limited to women admitted to hospital, rendering comparisons only possible after individual patient data meta‐analysis. It is very likely that the incidence of all infections was much higher in the UK, as reflected in the general population.^22^ Most other reported studies are facility‐based.^9^, ^23^ Population‐based registration studies into SARS‐CoV‐2 in pregnancy performed so far are all from members of the International Obstetric Survey System Network (INOSS). This highlights the importance of a registration system such as NethOSS, which enables rapid data collection, for instance in the case of a pandemic.

An increased risk of caesarean section after labour‐onset was demonstrated in SARS‐CoV‐2‐infected women, especially when they had symptoms at birth. Similar increases have been reported in the UK, Italy and New York City.^9^, ^23^, ^24^ This might be due to increased caution of the attending physician or presence of specific background characteristics such as high BMI and pre‐existing disease, which increase the risk of both SARS‐CoV‐2 infection and caesarean section. Neonatal outcomes in our study were reassuring and similar to results in other studies.^8^

To guide therapy and vaccination policies in the vulnerable group of pregnant women, especially subgroups at risk of severe disease, international individual patient data meta‐analysis based on robust population‐based data is warranted within INOSS, where in 17 countries uniform data on hospital‐admitted SARS‐CoV‐2‐positive pregnant women were collected. Long‐term consequences of SARS‐CoV‐2 infection for women and their babies remain unknown and information is urgently needed.^25^, ^26^, ^27^, ^28^, ^29^

## Conclusions

It is increasingly clear that pregnant women may comprise a vulnerable group in the COVID‐19 pandemic. In the Netherlands, not being of European country origin and being overweight or obese were risk factors for hospital admission. Infected women had higher odds of being induced or giving birth by caesarean section. Pregnant and postpartum women infected with SARS‐CoV‐2 appear to be at higher risk of hospital and ICU admission compared with SARS‐CoV‐2‐positive women in the same age group. Pregnant women should therefore be advised to adhere to social distancing and early testing and registration should be facilitated. Moreover, pregnant women with SARS‐CoV‐2 infection should be closely monitored, particularly in presence of additional risk factors, and long‐term follow‐up studies are warranted.

### Disclosure of interests

The authors report no conflict of interest.

### Contribution to authorship

EO and KB designed the study. EO wrote the first draft of the manuscript. EO and AR carried out the analyses. EO, AR, JZ, TS, TvdA and KB contributed to the development and conduct of the study. EO, AR, JZ, TV, TS, TvdA and KB contributed to interpretation of data, and edited and approved the final version of the article.

### Details of ethical approval

All procedures performed in studies involving human participants were in accordance with the ethical standards of the institutional and/or national research committee and with the 1964 Helsinki Declaration and its later amendments or comparable ethical standards. The NethOSS registration system is part of the National Perinatal Registry foundation in the Netherlands (Perined). This study did not require specific ethical approval and informed consent of participants was not obtained because Perined is allowed administrative permission in the Netherlands to access patient information from patient charts if the information used is not personally identifiable, concerns large numbers of participants and it is not feasible to trace and contact individual participants.

### Funding

No funding was received.

## Supporting information


**Figure S1**. Number of SARS‐CoV‐2‐infected pregnant women each week.

Supplementary Material

Supplementary Material

Supplementary Material

Supplementary Material

Supplementary Material

Supplementary Material

Supplementary Material

## References

[1] WHO . Coronavirus disease (COVID‐2019) situation reports. 2020 [https://www.who.int/emergencies/diseases/novel‐coronavirus‐2019/situation‐reports/]. Accessed 12 December 2020.

[2] Buekens P , Alger J , Bréart G , Cafferata ML , Harville E , Tomasso G . A call for action for COVID‐19 surveillance and research during pregnancy. Lancet Global Health 2020;8:e877–8. 32333854 10.1016/S2214-109X(20)30206-0 PMC7176390

[3] Liu H , Wang LL , Zhao SJ , Kwak‐Kim J , Mor G , Liao AH . Why are pregnant women susceptible to COVID‐19? An immunological viewpoint. J Reprod Immunol 2020;139: 103122. 32244166 10.1016/j.jri.2020.103122 PMC7156163

[4] Rac H , Gould AP , Eiland LS , Griffin B , McLaughlin M , Stover KR , et al. Common bacterial and viral infections: review of management in the pregnant patient. Ann Pharmacother 2019;53:639–51. 30556401 10.1177/1060028018817935

[5] Racicot K , Mor G . Risks associated with viral infections during pregnancy. J Clin Invest 2017;127:1591–9. 28459427 10.1172/JCI87490 PMC5409792

[6] Schwartz DA , Graham AL . Potential maternal and infant outcomes from (Wuhan) Coronavirus 2019‐nCoV infecting pregnant women: lessons from SARS, MERS, and other human coronavirus infections. Viruses 2020;12:194. 10.3390/v12020194 PMC7077337 32050635

[7] Galang RR , Chang K , Strid P , Snead MC , Woodworth KR , House LD , et al. Severe coronavirus infections in pregnancy: a systematic review. Obstet Gynecol 2020;136:262–72. 32544146 10.1097/AOG.0000000000004011 PMC7942856

[8] Allotey J , Stallings E , Bonet M , Yap M , Chatterjee S , Kew T , et al. Clinical manifestations, risk factors, and maternal and perinatal outcomes of coronavirus disease 2019 in pregnancy: living systematic review and meta‐analysis. BMJ 2020;370: m3320. 32873575 10.1136/bmj.m3320 PMC7459193

[9] Knight M , Bunch K , Vousden N , Morris E , Simpson N , Gale C , et al. Characteristics and outcomes of pregnant women admitted to hospital with confirmed SARS‐CoV‐2 infection in UK: national population based cohort study. BMJ 2020;369: m2107. 32513659 10.1136/bmj.m2107 PMC7277610

[10] Di Toro F , Gjoka M , Di Lorenzo G , De Santo D , De Seta F , Maso G , et al. Impact of COVID‐19 on maternal and neonatal outcomes: a systematic review and meta‐analysis. Clin Microbiol Infect 2021;27:36–46. 33148440 10.1016/j.cmi.2020.10.007 PMC7605748

[11] NVOG . Update registratie COVID‐19 positieve zwangeren in NethOSS. 2020 [https://www.nvog.nl/actueel/registratie‐van‐covid‐19‐positieve‐zwangeren‐in‐nethoss/] Accessed 12 December 2020.

[12] Schaap TP , Overtoom E , van den Akker T , Zwart JJ , van Roosmalen J , Bloemenkamp KWM . Maternal cardiac arrest in the Netherlands: a nationwide surveillance study. Eur J Obstet Gynecol Reprod Biol 2019;237:145–50. 31051417 10.1016/j.ejogrb.2019.04.028

[13] Schaap TP , van den Akker T , Zwart JJ , van Roosmalen J , Bloemenkamp KWM . A national surveillance approach to monitor incidence of eclampsia: the Netherlands Obstetric Surveillance System. Acta Obstet Gynecol Scand 2019;98:342–50. 30346039 10.1111/aogs.13493

[14] Unit NPE . [Internet]. 2020 [https://www.npeu.ox.ac.uk/assets/downloads/ukoss/forms/UKOSS_COVID‐19_v2.01_‐_27‐May‐2020_active_FINAL.pdf]. Accessed 27 November 2020.

[15] Goossen WT , Arns‐Schiere AM . Information architecture for perinatal registration in the netherlands. J Obstet Gynecol Neonatal Nurs 2017;46:310–21. 10.1016/j.jogn.2016.11.011 28089579

[16] National Institute for Public Health and the Environment MoH, Welfare and Sport, the Netherlands. Testing for COVID‐19. 2020. [https://www.rivm.nl/en/novel‐coronavirus‐covid‐19/testing‐for‐covid‐19]. Accessed 5 June 2020.

[17] RIVM RvVeM‐ . Epidemiologische situatie COVID‐19 in Nederland [Internet]. 2020 [https://www.rivm.nl/documenten/epidemiologische‐situatie‐covid‐19‐in‐nederland‐2‐april‐2020]. Accessed 02 April 2020.

[18] Hedermann G , Hedley PL , Bækvad‐Hansen M , Hjalgrim H , Rostgaard K , Poorisrisak P , et al. Danish premature birth rates during the COVID‐19 lockdown. Arch Dis Child Fetal Neonatal Ed 2021;106:93–5. 32788391 10.1136/archdischild-2020-319990 PMC7421710

[19] Philip RK , Purtill H , Reidy E , Daly M , Imcha M , McGrath D , et al. Unprecedented reduction in births of very low birthweight (VLBW) and extremely low birthweight (ELBW) infants during the COVID‐19 lockdown in Ireland: a ‘natural experiment’ allowing analysis of data from the prior two decades. BMJ Glob Health 2020;5:e003075. 10.1136/bmjgh-2020-003075 PMC7528371 32999054

[20] Docherty AB , Harrison EM , Green CA , Hardwick HE , Pius R , Norman L , et al. Features of 20 133 UK patients in hospital with covid‐19 using the ISARIC WHO Clinical Characterisation Protocol: prospective observational cohort study. BMJ 2020;369: m1985. 32444460 10.1136/bmj.m1985 PMC7243036

[21] Collin J , Byström E , Carnahan A , Ahrne M . Public Health Agency of Sweden’s Brief Report: pregnant and postpartum women with severe acute respiratory syndrome coronavirus 2 infection in intensive care in Sweden. Acta Obstet Gynecol Scand 2020;99:819–22. 32386441 10.1111/aogs.13901 PMC7273089

[22] Coronavirus COVID‐19 in the UK [Internet]. 2020 [https://coronavirus.data.gov.uk]. Accessed 27 November 2020.

[23] Maraschini A , Corsi E , Salvatore MA , Donati S , Group IC‐W . Coronavirus and birth in Italy: results of a national population‐based cohort study. Ann Ist Super Sanita 2020;56:378–89. 32959805 10.4415/ANN_20_03_17

[24] Prabhu M , Cagino K , Matthews KC , Friedlander RL , Glynn SM , Kubiak JM , et al. Pregnancy and postpartum outcomes in a universally tested population for SARS‐CoV‐2 in New York City: a prospective cohort study. BJOG 2020;127:1548–6. 32633022 10.1111/1471-0528.16403 PMC7361728

[25] Heneka MT , Golenbock D , Latz E , Morgan D , Brown R . Immediate and long‐term consequences of COVID‐19 infections for the development of neurological disease. Alzheimers Res Ther 2020;12:69. 32498691 10.1186/s13195-020-00640-3 PMC7271826

[26] Abboud H , Abboud FZ , Kharbouch H , Arkha Y , El Abbadi N , El Ouahabi A . COVID‐19 and SARS‐Cov‐2 infection: pathophysiology and clinical effects on the nervous system. World Neurosurg 2020;140:49–53. 32474093 10.1016/j.wneu.2020.05.193 PMC7255736

[27] Fraser E . Long term respiratory complications of covid‐19. BMJ 2020;370: m3001. 32747332 10.1136/bmj.m3001

[28] Salehi S , Reddy S , Gholamrezanezhad A . Long‐term pulmonary consequences of coronavirus disease 2019 (COVID‐19): what we know and what to expect. J Thorac Imaging 2020;35:W87–W9. 10.1097/RTI.0000000000000534 32404798

[29] Mongioì LM , Barbagallo F , Condorelli RA , Cannarella R , Aversa A , La Vignera S , et al. Possible long‐term endocrine‐metabolic complications in COVID‐19: lesson from the SARS model. Endocrine 2020;68:467–70. 32488837 10.1007/s12020-020-02349-7 PMC7266418

