# Supplementary figures and images for: SARS‐CoV‐2 infection in pregnancy during the first wave of COVID‐19 in the Netherlands: a prospective nationwide population‐based cohort study (NethOSS)
Source: BJOG. 2021 Sep 26;129(1):91–100. doi: 10.1111/1471-0528.16903 (PMC8652526; doi:10.1111/1471-0528.16903)

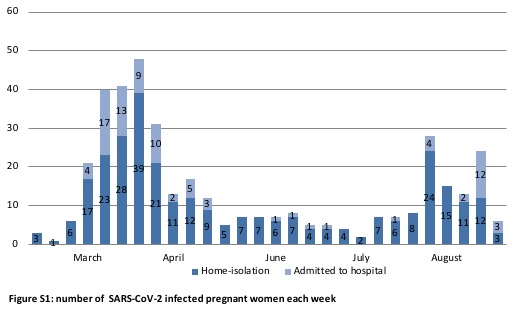

Supplement: Supplementary file 1 — Figure S1. Number of SARS‐CoV‐2‐infected pregnant women each week. [file BJO-129-91-s008.jpg]
